# Immediate postpartum long-acting reversible contraception in Ethiopia: A scoping review

**DOI:** 10.1371/journal.pone.0352352

**Published:** 2026-07-06

**Authors:** Ritbano Ahmed Abdo, Endalew Gemechu Sendo, Erdaw Tachbele Betre, Mohammed Sultan

**Affiliations:** 1 Department of Midwifery, Wachemo University, College of Medicine and Health Sciences, Hossana, Ethiopia; 2 School of Nursing and Midwifery, Addis Ababa University, College of Public Health, Addis Ababa, Ethiopia; 3 Department of Nursing, Addis Ababa University, College of Public Health, School of Nursing and Midwifery, Addis Ababa, Ethiopia; 4 Department of Statistics, Wachemo University, College of Natural and Computational Sciences, Hossana, Ethiopia; Mount Sinai School of Medicine: Icahn School of Medicine at Mount Sinai, UNITED STATES OF AMERICA

## Abstract

**Background:**

Immediate postpartum long-acting reversible contraception (IPP-LARC) is vital for reducing unintended pregnancies and improving maternal health. Despite extensive research in Ethiopia, evidence remains fragmented. The primary objective of this scoping review was to systematically map and synthesize the available evidence on IPP-LARC in Ethiopia and identify research gaps to inform future interventions.

**Methods:**

A scoping review was conducted following the JBI framework. Comprehensive searches were performed across PubMed, Cochrane Library, Hinari, Google Scholar, and grey literature sources. Studies reporting on IPP-LARC in Ethiopia were included. Data were charted and synthesized narratively. Two independent reviewers screened studies using Rayyan. Findings were presented in graphical, tabular, and narrative formats, adhering to PRISMA-ScR guidelines.

**Results:**

A total of 845 records were identified through database searches and other strategies, of which 42 studies met the inclusion criteria. Among these, 42 employed quantitative designs, two were implementation-focused quality improvement studies, one used a mixed-methods approach, and one was purely qualitative. Thematic focus varied: 16 studies assessed IPP-LARC uptake, 12 examined IPP-IUCD uptake, seven evaluated intention or acceptance of LARC, one evaluated IPP-IUCD acceptance and uptake, one assessed informed choice, one examined receipt of IPPFP counseling, one explored barriers and facilitators, and one focused on knowledge and attitude. Reported uptake of immediate postpartum family planning ranged from 20.0% to 53.2%, with variation influenced by socio-demographic, service-related, psychosocial, and relational factors.

**Conclusion:**

Evidence on IPP-LARC in Ethiopia highlights consistent determinants but is constrained by methodological and contextual gaps. Research remains dominated by cross-sectional, woman-centered studies, with limited attention to male engagement, community-level platforms, and qualitative insights. Addressing these gaps through rigorous, gender-inclusive, and context-sensitive intervention research is essential to strengthen shared decision-making and improve contraceptive uptake.

## Introduction

Preventing unintended and closely spaced pregnancies is a global priority for improving maternal and neonatal health outcomes [1–9]. The immediate postpartum period—defined as the time from childbirth up to 48 hours after delivery—represents a critical window of opportunity to initiate effective contraceptive methods [10]. During this period, women are often highly motivated to avoid pregnancy, they are in contact with health facilities, and long-acting reversible contraceptives (LARCs)—particularly implants and postpartum intrauterine devices (PP-IUDs)—can be safely provided before discharge [11,12]. Evidence indicates that immediate initiation of LARCs reduces unmet need for family planning, decreases unintended pregnancies, and prevents adverse maternal and neonatal outcomes [1,2,8,13]. Despite these benefits, uptake of immediate postpartum LARCs (IPP-LARCs) remains suboptimal in many low- and middle-income countries, including Ethiopia [14–16].

Evidence from Ethiopia demonstrates wide variability in the uptake of IPP-LARC methods, with overall uptake rates remaining low to moderate [17–22]. Meta-analyses estimate that the pooled prevalence of IPP-FP uptake is approximately 21% [16]. However, facility-based studies report figures ranging from below 3.3% to over 53.2%, depending on region, type of health facility, and method mix [17,19,20,23,24]. For example, research from Addis Ababa reported that about one-third of women initiated an IPP-LARC method [22], while studies from Jimma [23] and other health facilities indicated even lower uptake, particularly of IUCD [18,25]. Such heterogeneity underscores contextual, methodological, and programmatic differences across Ethiopian health facilities.

Several determinants of IPP-LARC uptake have been identified in the Ethiopian context. Positive predictors include adequate antenatal counseling about postpartum contraception, receipt of family planning counseling during antenatal care visits, good knowledge and favorable attitudes toward LARC methods, and joint decision-making with partners [19,22,23,25]. Conversely, barriers to uptake include provider skill gaps in postpartum insertion techniques, inconsistent availability of commodities, weak integration of family planning (FP) with maternal health services, and prevailing misconceptions or fear of side effects, male partner refusal, and the appropriateness of LARCs [26–28]. Encouragingly, quality improvement interventions in some facilities have shown promise, suggesting that service delivery innovations and health system strengthening can substantially increase utilization when properly implemented [29,30].

From a policy perspective, Ethiopia has recognized postpartum family planning as an essential health service and has promoted its integration into maternal and newborn care [31,32]. However, despite strong national commitments, implementation remains uneven across regions and facility levels [16,28,33–36]. Operational challenges—including inadequate provider training, supply chain interruptions, and weak coordination between delivery and FP units—continue to undermine efforts to scale up IPP-LARC nationwide [28]. This discrepancy between policy intentions and practice highlights the need for comprehensive evidence to guide strategies that strengthen postpartum contraceptive services.

The existing literature on IPP-LARC in Ethiopia is fragmented, comprising cross-sectional surveys, facility-based studies, and a few systematic reviews, each reporting outcomes on different methods, timeframes, and determinants [20–22,33,36,37]. Evidence gaps remain, particularly regarding rural and primary health care settings, provider- and system-level interventions, and women’s perspectives on acceptability and continuation [38,39]. Given these gaps and the heterogeneity of findings, a scoping review is warranted to map the breadth of available evidence, identify research and implementation gaps, and guide policymakers and program managers toward more effective strategies for expanding access to IPP-LARC in Ethiopia.

## Objectives of the review

The primary objective of this scoping review is to systematically map and synthesize the available evidence on IPP-LARC in Ethiopia. Specifically, the review aims to identify the extent, range, and nature of published and unpublished studies addressing various dimensions of IPP-LARC, including uptake, acceptance, intention, and knowledge among different population groups such as women, male partners, adolescents, healthcare providers, and other stakeholders. In addition, the review seeks to examine publication trends, study designs, and geographic distribution of research, as well as summarize reported patterns of IPP-LARC uptake and related behavioral outcomes. Furthermore, it will explore client-level and provider/system-level factors that act as facilitators or barriers to IPP-LARC provision and uptake. Finally, the review will identify gaps in the existing literature—such as underrepresented populations, unstudied outcomes, and lack of specific study types like qualitative research, economic evaluations, and long-term follow-up—to inform future research priorities and guide programmatic and policy interventions.

## Method

### Design

We utilized a scoping review following the Joanna Briggs Institute (JBI) framework to map and synthesize evidence on IPP-LARC in Ethiopia. The JBI framework involves six steps: (1) identifying the research question, (2) identifying relevant studies, (3) selecting studies based on eligibility criteria, (4) charting the data using a standardized form, (5) collating, summarizing, and reporting the results, and (6) consultation with stakeholders (optional). Scoping reviews are appropriate for exploring emerging topics with heterogeneous evidence, aiming to describe the scope and nature of available literature rather than assess study quality or estimate effect sizes [40]. The review focused on documenting patterns of IPP-LARC uptake, determinants, barriers, facilitators, and policy influences within the Ethiopian context. A pre-registered protocol guided the process (Open Science Framework, OSF: [https://osf.io/wh5jn], registered on October 12, 2025) [41].

### Eligibility criteria

This scoping review adhered to the Population–Concept–Context (PCC) framework as recommended by the JBI [42].

### Population

The review targeted studies involving postpartum women within 48 hours of childbirth, including those delivering in health facilities and eligible for contraceptive services. In addition, evidence related to pregnant women, adolescents, male partners, and healthcare providers such as midwives, nurses, and physicians involved in counseling and LARC provision was considered. The review also included studies addressing knowledge, attitudes, and intentions regarding IPP-LARC among these groups, as well as health system factors such as policies, service delivery structures, and commodity availability that influence implementation and uptake.

### Concept

The core concept of this review is IPP‑LARC, explored through multiple outcome measures including uptake (prevalence), intention, acceptance, knowledge, attitudes, and determinants. To ensure a comprehensive mapping of the Ethiopian landscape, studies were included if they focused on the overarching category of IPP‑LARC (combined IUCD and implants) or specifically on its individual components (IPP‑IUCD alone or IPP‑implants alone).

In addition, studies addressing broader immediate postpartum family planning (IPPFP) were considered only if they provided disaggregated or method-specific data relevant to LARC methods (i.e., implants and/or IUCDs). Studies reporting only overall postpartum contraceptive use without distinguishing LARC methods were excluded. This approach was adopted to ensure conceptual alignment with the focus on IPP‑LARC while allowing inclusion of relevant evidence embedded within broader postpartum family planning research.

Studies addressing facilitators (e.g., antenatal counseling, provider attitudes), barriers (e.g., skill gaps, stockouts, cultural misconceptions), and implementation aspects such as service integration, training, counseling quality, and user/provider perspectives were included.

### Context

The review focused on Ethiopia, covering all levels of healthcare—primary (health posts, centers), secondary (general hospitals), and tertiary (referral hospitals). Articles were considered within Ethiopia’s socio-cultural, economic, and policy environment, accounting for regional disparities and operational challenges.

### Types of evidence sources

This review included primary studies employing quantitative, qualitative, and mixed-methods designs. Eligible study types comprised cross-sectional studies, cohort studies (prospective and retrospective), case-control studies, interventional studies, and qualitative research. Systematic reviews and relevant grey literature were also included. Conference abstracts were considered only if they provided sufficient data for extraction. Editorials, commentaries, and opinion pieces were excluded.

In line with the focus of this review, studies examining IPPFP were included only when they reported disaggregated findings specific to long-acting reversible contraceptive methods (e.g., implants or intrauterine contraceptive devices). Studies presenting aggregate postpartum contraceptive utilization without method-specific differentiation were excluded to maintain conceptual consistency with IPP‑LARC.

### Information sources and Search strategy

A structured three-step search strategy was employed in this scoping review to identify relevant literature. Initially, a preliminary search of PubMed and Google Scholar was conducted to identify pertinent keywords and controlled vocabulary terms. These terms informed a comprehensive and systematic search across major electronic databases, including PubMed/MEDLINE, Hinari, and the Cochrane Library. The PubMed search strategy included terms such as“postpartum”, “long-acting reversible contraceptives”, uptake, “use” “utilization”, “acceptance” “LARCs”, “intrauterine contraceptive device”, “IUCD”, “implant” “immediate postpartum”, and “Ethiopia” and Boolean operators (AND, OR) used for accuracy. This strategy was subsequently adapted to align with the syntax and subject headings specific to each database. In addition to database searches, grey literature was explored by scanning Google Scholar, African Journals Online, and reviewing ProQuest for theses and dissertations. To ensure thoroughness, the reference lists of all included studies were manually reviewed to identify additional relevant sources.

The search was limited to studies published in English from January 01, 2010, to October 12, 2025,“ or “between January 01, 2010, and October 12, 2025, reflecting the contemporary policy landscape surrounding IPP-LARC. The focus was placed on evidence related to uptake patterns, influencing factors, provider perspectives, and programmatic implementation. (The detailed search strategy is provided in S1 File)

### Study selection

All identified citations were imported into Rayyan, which facilitated blinded collaboration, duplicate removal, and screening. After a pilot screening, titles and abstracts were independently reviewed by two reviewers based on predefined inclusion criteria. Inter-rater reliability during the screening phase was assessed using the percentage of agreement, yielding an initial agreement rate of 98% between the two reviewers. Sources deemed potentially relevant were retrieved in full text and screened in Rayyan. All included article references were exported to EndNote (version 21) for reference management and citation formatting. Full-text articles were assessed by two independent reviewers, and any discrepancies were resolved through discussion until 100% consensus was achieved. Reasons for exclusion at the full-text stage were systematically documented. The overall study selection process—including the number of records identified, screened, included, and excluded—was transparently presented using a PRISMA-ScR flow diagram to ensure clarity and reproducibility [43].

### Data charting

Data from the included studies were systematically extracted using a structured template aligned with the review objectives. The extraction process captured key characteristics such as author(s), year of publication, objective, geographic region, study design, prevalence of IPP-LARC by type (implant plus IUCD), IUCD only and implant only, timing of measurement, data collection tools, and study population details, including participant demographics and type of health facility. Additionally, outcomes, key findings, recommendations, and reported limitations were documented. The data extraction tool was iteratively refined throughout the review process to ensure completeness and accuracy. Where necessary, corresponding authors were contacted to obtain missing or additional information.

### Data analysis and presentation

A descriptive narrative synthesis was conducted to organize and interpret the extracted data. Findings were presented using narrative text, tables, and figures to illustrate the scope, distribution, and thematic patterns of evidence on IPP-LARC in Ethiopia. In line with scoping review methodology, a formal critical appraisal of included studies was not conducted. The purpose of this review was to map the extent, range, and characteristics of available evidence on IPP‑LARC in Ethiopia rather than to evaluate methodological quality or risk of bias. Therefore, all studies meeting the inclusion criteria were included regardless of quality. However, key methodological characteristics such as study design, setting, sample size, and measurement timing were systematically reported to allow readers to interpret findings within their methodological context.

### Ethics and dissemination

As this scoping review exclusively utilized publicly available data, ethical approval was not required. The findings of the review have been submitted for publication in a peer-reviewed journal. In addition to academic dissemination, the results will be shared through presentations at relevant national and international conferences. A policy brief summarizing key findings and recommendations will also be prepared and disseminated to the Ethiopian Federal Ministry of Health and other key stakeholders to inform programmatic and policy-level decision-making.

## Results

### Search result

We identified 846 articles from multiple electronic databases and supplementary sources: PubMed/MEDLINE (n = 291), Hinari (n = 472), the Cochrane Library (n = 8), Google Scholar (n = 69), African Journals online (n = 4), and citation tracking (n = 2). After merging all records, 263 duplicates were removed using Rayyan, resulting in 494 unique articles for title and abstract screening. Of these, 89 articles were deemed potentially relevant and retrieved for full-text review. Following full-text assessment, 47 studies were excluded, primarily because they did not report outcomes related to IPP-LARC uptake, intention, knowledge, or associated determinants. Ultimately, 42 studies met the eligibility criteria and were included in the final scoping review. The review process was documented in a flowchart following the PRISMA-Sc guidelines (S1 Fig).

### Characteristics of the included studies

Among the studies included in this review, the majority were cross-sectional designs (n = 33) [17–25,33,35–39,44–60]. Other study types comprised three case-control studies [61–63], one quasi-experimental study [64], one continuous quality improvement study [29], one pre–post intervention study [65], one mixed-methods (pre-post intervention) study [66], one qualitative study [67], and one systematic review and meta-analysis [68]. Geographically, Oromia was most frequently represented (n = 14) [17,21,23,29,36–39,45,47,50,51,54,64], followed by Addis Ababa (n = 10) [19,22,44,46,48,52,60,65,66,69], Amhara (n = 6) [24,55,58,59,61,62], Southern Ethiopia (n = 4), Central Ethiopia region (n = 1), Sidama regional state (n = 4), while three studies were multi-regional or national in scope [18,56,68] and Tigray and Afar appeared only in multi-region analyses. Publication trends indicate a sharp increase in research output, with eight studies published between 2010 and 2020 [24,35,44,46,50,52,63,64] and 34 between 2021 and 2025 [17–23,25,29,33,36–39,45,47–49,51,53–62,65–69]. Most primary studies (n = 37) were institution-based (public hospitals = 21, public health facilities = 15 and two health center), while only two were community-based and none were conducted in health posts [18,25]. Commonly assessed outcomes included IPP-LARC (implant plus IUCD) [18,20–23,25,29,37,44,46,52,58,60,61,66] and IUCD only [17,19,35,47,51,55,57,62–64,69], alongside intention or acceptance among pregnant or postpartum women [24,38,45,48–50,53,54,57]; knowledge and attitude [59], whereas informed choice and counseling receipt were assessed in only one study each [33,56]. One single study also assessed acceptance and uptake of IPP-IUCD [57].

The primary study populations comprised postpartum women who had delivered within the preceding 48 hours, with recruitment predominantly occurring in health facilities, including public hospitals and primary healthcare facilities. A smaller subset of studies focused on pregnant women attending ANC services to assess intention, knowledge, and attitudes. Male partners were rarely included as direct participants. Notably, only one qualitative study [67] explicitly incorporated husbands, adolescents, and health system stakeholders, whereas other studies referenced “partner support” solely as a variable reported by women respondents [19–22,36,49,58,60]. Regarding the timing of data collection or measurement, among the articles that assessed the uptake and acceptance of the method, data were collected in most cases before discharge or within 48 hours. Only two studies collected data retrospectively (one within 6 months and one within 12 months) (Table 1). For further details, see S1 Table, which presents the author name, publication year, study area, design, sample size, population, outcome variables, key findings, recommendations, and limitations of each included study.

**Table 1:** Characteristics of included studies on IPP-LARC in Ethiopia.

Characteristic		Frequency
Study type	Quantitative	40
	Qualitative	1
	SR & MA	1
Specific designs	Cross-sectional	33
	Case-control	3
	Quasi-experimental/pre-post study design	3
	Quality improvement intervention study	1
	Qualitative	1
	SR & MA	1
Geographic distribution	Oromia	14
	Addis Ababa	10
	Amhara	6
	Sidama region	4
	Central Ethiopia region	1
	Southern Ethiopia	4
	Multi-region/National	3
Publication years (including SR and MA)	2010–2020	8
	2020–2025	34
Study Setting (Primary study only)	Institution-based	39
	Community-based	2
Outcomes assessed (primary studies only)	IPP-LARC(Implant + IUCD) utilization	16
	IPP-IUCD utilization	12
	Intention/acceptance of IUCD only	7
	Barriers and facilitators	1
	IPP-IUCD acceptance & Utilization	1
	IPP-LARC intention	1
	Knowledge and attitude	1
	IPP-LARCs informed choice	1
	Receipt of IPPFP counseling	1
Data collection tools (Primary study only)	Structured questionnaires	33
	Semi-structured questionnaires	4
	IDIs, KIIs, FGDs	1
	Logbooks, charts, interviews, checklists, audits	1
	SQ, interview guides, record review	1
	Structured check list	2

### IPP-LARC uptake

Research on IPP-FP in Ethiopia spans the continuum from demand generation to actual uptake. Studies assessing the uptake of IPP-LARC reported utilization rates ranging from 20.0% to 53.2% [23,58]. variation reflects differences across study settings, facility types, geographic areas, contraceptive methods assessed, and period of study, as further detailed in the stratified analysis below [19,22–25,33,39,44,46,54,61–63,69]. One systematic reviews and meta-analyses estimated pooled prevalence at 8.37% for IPP-IUCD [68]; however, numerous primary studies have been conducted subsequent to this review [17,25,37–39,53,54,61,69].

### Acceptance and Intention, knowledge, and attitude

Investigations on acceptance and intention—primarily among pregnant women—reported higher intention rates (34.9%–37.6%) [36,45], indicating a gap between intention and uptake. Several studies examined knowledge and attitude, identifying adequate knowledge and positive attitudes as predictors of acceptance and utilization [17,19,21,36,38,51,60,61]. Nevertheless, only one study explicitly evaluated knowledge as the primary outcome [59], reporting that 36% of women had good knowledge about IUCD and 48.7% had a positive attitude toward IUCD. Provider knowledge was not measured as a primary outcome in the included studies [29,30,66] (S1 Table).

### Stratified patterns of IPP-LARC uptake

The evidence regarding IPP-LARC uptake in Ethiopia reveals distinct structural variations when stratified by facility level, geography, method, and study design. Utilization was highest in public hospitals (20%–53.2%) [23,58] compared to the moderate-to-lower ranges seen in public health centers (9.9%–30.7%) [22,46].

Geographically, Oromia reported the highest prevalence of IPP-LARC (up to 53.2%) [23], while Addis Ababa and the Southern regions showed more moderate levels. Disaggregation by method indicates that implants (5.7%–47.4%) [21,23] generally outpaced IUCDs (1.9%–27.2%) [37,51]. Furthermore, study design influenced outcomes; facility-based cross-sectional studies often documented higher rates than community-based surveys. Finally, a temporal trend is evident: studies published between 2017 and 2020 typically reported utilization below 25%, while research from 2022 to 2025 frequently documented uptake exceeding 30%, reflecting a positive evolution in service adoption.

### Behavior gap and theoretical framework

Across the included studies, a consistent discrepancy was observed between intention, acceptance, and actual uptake of IPP‑LARC. Intention to use IPP‑LARC, primarily measured among pregnant women during ANC, ranged from 34.9% to 37.6% [36,45], whereas acceptance levels in facility-based studies ranged from 9.9% to 35.6% [49,54], and actual utilization in several studies remained lower than acceptance levels. This pattern indicates a clear behavioral gap between expressed intention and actual uptake of IPP‑LARC.

Determinants identified across studies—including knowledge, attitude, partner involvement, counseling exposure, and reproductive intentions—were reported to influence both intention and acceptance, as well as transition to actual use. These findings align with behavioral models that distinguish between intention and actual behavior, where additional barriers at the individual, social, and health system levels influence the translation of intention into practice.

#### Determinants of IPP-LARC/IUCD Uptake.

Socio-demographic and economic characteristics exert a significant influence on IPP-LARC uptake. Older maternal age (typically 25–34 years and above) and higher educational attainment consistently predict greater uptake [22,51,55]. Employment status and household wealth also play critical roles; women employed in government positions or belonging to wealthier households exhibit higher odds of uptake, whereas housewives demonstrate lower uptake [19,25,38]. Women’s empowerment emerges as a strong enabling factor, reinforcing the importance of autonomy in reproductive health decisions [25].

Service-related determinants are among the most influential predictors. Receipt of counseling during ANC, delivery, or the postpartum period is consistently associated with increased uptake across nearly all studies [22,23,25,33,36,61]. Quality of care, including respectful maternity services, further enhances uptake, while experiences of disrespect or abuse act as significant barriers [21]. Structural enablers such as health facility delivery, availability of maternity waiting homes, and uninterrupted commodity supply also facilitate uptake [18,29]. Provider training and dedicated counseling services amplify these effects, highlighting the critical role of health system readiness [29,65].

Knowledge and psychosocial factors remain central to uptake. Adequate knowledge and favorable attitudes toward LARC methods strongly predict uptake, whereas fear of side effects constitutes the most frequently cited barrier [17,19,22,38,39,51,55,57,60,61]. Partner-related dynamics further shape outcomes; spousal support, joint decision-making, and partner involvement in counseling sessions significantly increase uptake, while husband opposition remains a major deterrent [19,21,22,37]. Finally, reproductive history—including higher parity, desire to limit or space births, cesarean delivery, frequent ANC attendance, and prior LARC use—consistently correlates with greater uptake [17,23,39,47,51].

#### Determinants of intention to use IPP-LARC/IUCD.

Determinants of intention largely mirror those influencing actual uptake, reflecting the anticipatory decision-making process during pregnancy. Socio-demographic factors such as advanced maternal age, higher education, and improved economic status positively influence intention [45,53]. Knowledge and attitude remain critical, with well-informed women and those holding favorable perceptions demonstrating stronger intentions. Reproductive history, including multiparity and prior LARC use, further predicts readiness [36]. Psychosocial constructs derived from the Theory of Planned Behavior—attitude, subjective norms, and perceived behavioral control—emerge as direct predictors of intention, underscoring the role of behavioral theory in understanding contraceptive decision-making [53].

#### Determinants of acceptance of IPP-LARC/IUCD.

Acceptance, often conceptualized as readiness to use if offered, shares nearly identical determinants with intention and uptake. Counseling, positive attitudes, partner discussion, and multiparity consistently emerge as strong predictors [48,57]. Additional enabling factors include higher education, greater income, and completion of ANC follow-ups [48]. Conversely, barriers to acceptance predominantly revolve around fear of side effects, lack of awareness, spousal opposition, and preference for alternative methods [38].

#### Interventions to enhance IPP-LARC Uptake.

Few intervention studies have demonstrated promising results. Sori et al. applied a Continuous Quality Improvement (CQI) strategy using the Plan–Do–Study–Act (PDSA) cycle, which increased uptake from 6.9% at baseline to 25.4% post-intervention [29]. Wayessa et al. implemented focused family planning counseling based on the Health Belief Model, which significantly improved IPP-IUCD uptake from 4.8% to 12.4% [64]. Tesfaye et al. tested a multi-component package of interventions—including provider training and the provision of private counseling spaces—resulting in an increase in uptake from 65.9% to 72.3% and improved service quality [66]. Similarly, Sium et al. demonstrated that assigning a dedicated resident or staff member specifically for postpartum family planning counseling increased uptake from 15.4% to 20.4% [65]. The evidence indicates that structured, theoretically-informed interventions are effective at increasing uptake. However, significant heterogeneity in intervention designs (ranging from provider-side training to client-focused behavioral models) and variation in outcome measurement periods precluded a systematic comparison of effect sizes. This diversity emphasizes the need for tailored strategies rather than a single standardized approach (S2 Table).

#### Research gaps identified.

The scoping review revealed several critical gaps in the existing literature on IPP-LARC uptake in Ethiopia. Methodologically, most studies were cross-sectional and institution-based, which limits causal inference and generalizability to community settings. Intervention studies were scarce, and those available lacked control groups and rigorous designs, reducing the strength of evidence. Heavy reliance on self-reported data introduced recall and social desirability bias, while short counseling-to-decision intervals and absence of follow-up restricted assessment of long-term outcomes such as continuation, satisfaction, and side effects. Furthermore, qualitative research was almost absent, with only one qualitative and one mixed-method study identified, leaving significant gaps in understanding sociocultural norms, gender dynamics, and health system barriers

Geographical representation was another limitation, as most studies were concentrated in urban or facility-based settings, neglecting rural areas and primary health care platforms such as Health Posts, which are the most accessible level of care in Ethiopia. Knowledge gaps were also evident: only one study assessed women’s knowledge of IPP-LARC, and no study examined male knowledge, despite men’s strong influence on contraceptive decision-making in Ethiopia’s patriarchal context. Programmatically, most interventions focused on maternal and child health counseling directed at women, excluding men from FP discussions. Critically, no study has evaluated an integrated approach that combines partner support and high-quality counseling within a theory-driven framework, nor measured intermediary behavioral outcomes such as couple communication, self-efficacy, and shared decision-making.

### Directions suggested by included studies

The included studies consistently recommended strengthening counseling during antenatal, delivery, and postpartum periods and integrating FP into routine maternal and child health services. Addressing misconceptions through targeted education and promoting male involvement were emphasized as key strategies to improve uptake. Community awareness campaigns and initiatives to empower women through education and economic opportunities were also highlighted. At the provider and system level, recommendations included training health workers on insertion techniques and counseling skills, ensuring consistent availability of FP commodities, and implementing quality improvement models supported by audit-feedback mechanisms. Policy-level directions focused on scaling up integrated FP interventions, such as maternity waiting homes and dedicated counseling staff, and conducting qualitative research to explore cultural barriers and informed choice. Operational studies were also recommended to evaluate cost-effectiveness and sustainability of integrated postpartum FP programs.

## Discussion

This review synthesized evidence from 42 studies on IPP-LARC in Ethiopia, highlighting key trends, gaps, and determinants associated with uptake and intention. The findings reveal substantial progress in research output, particularly in the last five years, but also underscore persistent methodological, geographic, and thematic limitations that constrain the effectiveness of IPP-LARC programs.

Most included studies employed quantitative methods (predominantly cross-sectional), with only a limited number adopting experimental, qualitative, or mixed-method approaches. While cross-sectional studies provide valuable prevalence estimates, they offer limited insight into causal relationships and the socio-cultural and behavioral dynamics related to contraceptive uptake

The limited number of qualitative and mixed-method studies—comprising two CQI studies, one pre–post intervention, and one qualitative study—restricts the exploration of contextual factors such as gender norms, provider–client interactions, and decision-making processes. Future research could benefit from increased use of qualitative and mixed-method designs to better capture these relational and cultural determinants relevant to context-specific interventions. These patterns indicate the need for a broader range of study designs, including longitudinal and implementation research, to inform the development of scalable interventions [29,30,64].

Research was disproportionately concentrated in Oromia and Addis Ababa, with limited representation from regions such as Sidama, Central Ethiopia, and Southern Ethiopia [20,24,25,33,35,53,54]. Tigray and Afar were included only in multi-regional analyses [18]. No studies were identified in Somalia, Dire-Dawa, Benishangul, Gambela, or the Southwest Ethiopia region. These patterns indicate limited evidence from several regions, suggesting gaps in geographic coverage. Furthermore, most studies were institution-based, primarily in public hospitals, while community-level evidence remains scarce. The absence of studies in health posts—where a significant proportion of rural births occur—suggests a disconnect between research and service realities in Ethiopia’s primary healthcare system.

The overwhelming focus on postpartum women recruited after delivery reflects the clinical orientation of IPP-FP research [17,19,23,38,46–52,61,62,69]. Pregnant women attending ANC services were included in a smaller subset of studies, primarily to assess intention and knowledge [36,49,53,59]. Male partner involvement was limited across studies. Only one study explicitly included male participants [67]. Evidence from included studies suggests partner support is associated with uptake [22,23,25,37], but few studies assessed knowledge or attitudes at the couple level. Future research should incorporate couple-level knowledge and behavioral indicators to design interventions that foster joint responsibility and informed choice.

IPP-LARC uptake rates varied widely (3.3%–55.8%), with variation observed across facility type, counseling exposure, and socio-demographic characteristics. Systematic reviews estimated pooled prevalence at 8.37% for IPP-IUCD [68], yet recent primary studies suggest evolving patterns post-2022. Intention rates among pregnant women (34.9%–37.6%) consistently exceeded actual uptake [36,45], highlighting a persistent intention–behavior gap. Knowledge and positive attitudes emerged as strong predictors of both intention and utilization, yet only one study assessed knowledge as a primary outcome [59], and none evaluated provider knowledge systematically. This gap underscores the need for interventions that target both client and provider knowledge, as well as couple communication, to bridge the intention–behavior divide.

Determinants of IPP-LARC uptake were multifaceted, spanning socio-demographic, economic, service-related, and psychosocial domains. Higher maternal age, education, and household wealth were frequently associated with uptake, while counseling during ANC, delivery, or postpartum was commonly reported in relation to higher uptake. Structural factors such as health facility delivery, availability of maternity waiting homes, and uninterrupted commodity supply were also commonly reported in relation to higher uptake. Conversely, disrespectful care and stock-outs were reported as barriers. For intention, constructs consistent with the Theory of Planned Behavior—attitude, subjective norms, and perceived behavioral control—were frequently associated with intention to use IPP-LARC.

A limited number of intervention studies were identified, including CQI strategies, focused counseling models, and multi-component approaches integrating provider training and commodity management [29,64–66]. These studies reported increases in IPP-LARC uptake following implementation, although the magnitude of change varied across studies. Few studies assessed combined approaches addressing multiple determinants, such as partner involvement and counseling, within a theory-informed framework. Further research using implementation science approaches may help to assess the applicability, scalability, and sustainability of these intervention strategies across different settings.

Overall, the literature identifies multiple determinants of IPP‑LARC uptake, intention, and acceptance; however, the evidence is largely derived from cross-sectional studies, with limited evaluation of intervention approaches. Additional research using diverse methodological designs may help to better understand how these factors operate across contexts.

## Strengths and limitations

This scoping review has several strengths. A comprehensive search strategy across multiple databases and grey literature sources ensured broad coverage of relevant studies, while the inclusion of diverse study designs allowed mapping of the full scope of evidence on IPP-LARC uptake and related constructs. Furthermore, the identification of consistent determinants and critical gaps provides a strong foundation for future intervention research. However, certain limitations should be acknowledged. Consistent with scoping review methodology, no formal quality appraisal was conducted, which may limit interpretation of methodological rigor. However, detailed reporting of study characteristics was used to support contextual interpretation of findings.

### Policy and programmatic implications

The findings have significant implications for FP programs and health system strategies in Ethiopia and similar contexts. First, strengthening counseling services during ANC and delivery should be prioritized, as counseling consistently predicts uptake. Second, policies should actively promote male partner involvement in reproductive health decision-making, moving beyond passive acknowledgment to structured engagement. Third, integrating multi-session counseling into the ANC platform at health posts can enhance accessibility and continuity of care, particularly for rural populations. Fourth, investments in provider training, commodity availability, and respectful maternity care are essential to create an enabling environment for IPP-LARC uptake. Fifth, programmatic monitoring should incorporate behavioral indicators—such as couple communication, self-efficacy, and shared decision-making—to track progress toward sustained contraceptive use. Finally, research and program design must explicitly include qualitative approaches to capture socio-cultural norms, gender dynamics, and provider-client interactions, and systematically assess knowledge among both women and men, ensuring interventions are informed by contextual realities.

## Conclusion

This scoping review demonstrates that although determinants of IPP-LARC uptake—such as counseling, partner support, and structural factors—are well-documented, the evidence base remains dominated by cross-sectional, woman-centered studies conducted primarily in hospital settings. Male involvement is minimal, interventions rarely leverage the antenatal period, and health posts—the most accessible platform for rural populations—are largely overlooked. Moreover, behavioral mediators such as couple communication and self-efficacy are seldom assessed, limiting understanding of mechanisms that drive uptake. Critically, qualitative research and systematic measurement of both partners’ knowledge and attitudes are almost absent, leaving gaps in understanding relational and cultural influences. Addressing these gaps through context-specific, gender-inclusive strategies and rigorous, theory-driven intervention research—including qualitative and mixed-method designs—is essential to promote shared decision-making and improve contraceptive use in Ethiopia.

### Future research agenda.

To address the critical gaps identified in IPP-LARC research in Ethiopia, future studies should prioritize the following areas:

**1. Rigorous intervention trials**

Move beyond observational designs to implement randomized or quasi-experimental studies that test structured, theory-driven interventions aimed at improving IPP-LARC uptake.

**2. Couple-based approaches**

Design and evaluate multi-session antenatal counseling interventions that actively involve male partners, delivered through health posts and other primary health care platforms to enhance accessibility and shared decision-making.

**3. Behavioral and knowledge outcomes**

Incorporate comprehensive measurement of behavioral mediators—including knowledge, attitudes, couple communication, and self-efficacy—for both women and men to understand pathways of effect and improve intervention targeting.

**4. Qualitative and mixed-method research**

Conduct in-depth qualitative studies to explore socio-cultural norms, gender dynamics, and provider-client interactions that influence contraceptive decision-making. Mixed-method designs should complement quantitative trials to capture contextual factors and implementation challenges.

**5. Longitudinal designs**

Implement follow-up studies to assess sustainability of IPP-LARC use, continuation rates, satisfaction, and side effects over time, addressing the current gap in long-term outcome data.

**6. Health System and Community-Level Evidence**

Expand research beyond hospital settings to include health posts and community platforms, where most rural births occur, ensuring findings are generalizable to Ethiopia’s primary health care context.

## Supporting information

S1 File Search terms summary. (DOCX)

S1 Fig PRISMA 2020 flow diagram showing study selection process. (TIF)

S1 Table Characteristics of included studies on immediate postpartum family planning in Ethiopia. (DOCX)

S2 Table Comparison of IPP-LARC uptake in intervention studies. (DOCX)
