# Supplementary material for: Immediate postpartum long-acting reversible contraception in Ethiopia: A scoping review
Source: PLoS One. 2026 Jul 6;21(7):e0352352. doi: 10.1371/journal.pone.0352352 (PMC13336211; doi:10.1371/journal.pone.0352352)
Supplement: S1 File — (DOCX) [file pone.0352352.s001.docx]

**S1File. Search terms summary**

| **Database** | **Search** | **result** |
| --- | --- | --- |
| **PubMed** | (("Postpartum Period"[MeSH Terms] OR "postpartum"[Text Word] OR "postnatal"[Text Word] OR "puerperium"[Text Word] OR "after childbirth"[Text Word] OR "after delivery"[Text Word] OR "immediate postpartum"[Text Word]) AND ("Long-Acting Reversible Contraception"[MeSH Terms] OR "long-acting reversible contraceptive"[Text Word] OR "LARC"[All Fields] OR "intrauterine device"[Text Word] OR "IUD"[Text Word] OR "IUCD"[Text Word] OR "implant"[Text Word] OR "contraceptive implant"[Text Word] OR "Implanon"[Text Word] OR "Jadelle"[Text Word] OR ("Family Planning Services"[MeSH Terms] OR "family planning"[Text Word] OR "contraception"[Text Word] OR "maternal health"[Text Word] OR "health facility"[Text Word] OR "primary health care"[Text Word])) AND ("uptake"[Text Word] OR "utilization"[Text Word] OR "use"[Text Word] OR "adoption"[Text Word] OR "acceptance"[Text Word] OR "coverage"[Text Word] OR "service uptake"[Text Word] OR "contraceptive use"[Text Word] OR "contraceptive adoption"[Text Word]) AND ("Ethiopia"[Text Word] OR "Ethiopia"[MeSH Terms]) AND ("loattrfree full text"[Filter] AND "medlinestatus medline"[All Fields] AND "loattrfull text"[Filter] AND "humans"[MeSH Terms] AND 2010/01/01:2025/12/12[Date - Publication] AND "english"[Language]) AND ("loattrfree full text"[Filter] AND "medlinestatus medline"[All Fields] AND "loattrfull text"[Filter] AND "humans"[MeSH Terms] AND 2010/01/01:2025/12/12[Date - Publication] AND "english"[Language]) AND ("loattrfree full text"[Filter] AND "medlinestatus medline"[All Fields] AND "loattrfull text"[Filter] AND "humans"[MeSH Terms] AND 2010/01/01:2025/12/12[Date - Publication] AND "english"[Language])) AND ((medline[Filter]) AND (fft[Filter]) AND (2010/1/1:2025/10/18[pdat]) AND (english[Filter])) | **291** |
| **Hinari** | ("immediate postpartum" OR postnatal OR postpartum) AND ("long-acting reversible contraceptive" OR LARC OR implant OR IUD OR IUCD OR Implanon OR Jadelle OR "family planning services" OR "maternal health" OR "primary health care") AND (uptake OR use OR utilization OR adoption OR acceptance) AND (Ethiopia): Filter: Full text online, Journal article, English and 2010/01/01:2025/12/12[Date - Publication] | **472** |
| **Google*** | ("immediate postpartum" OR postnatal OR postpartum) AND ("long-acting reversible contraceptive" OR LARC OR implant OR IUD OR IUCD OR Implanon OR Jadelle) AND (uptake OR use OR utilization OR adoption OR acceptance) AND (Intention) AND (Knowledge) AND (Ethiopia) | **69** |
| African journal online | Immediate postpartum long-Acting reversible contraceptive in Ethiopia | **4** |
| Citation search | | **2** |
|  | Total | **847** |

**Cochrane library**

**Date Run: 18/11/2025 17:34:58**

| **ID** | **Search** | **Result** |
| --- | --- | --- |
| #1 | Postpartum | 16910 |
| #2 | MeSH descriptor: [Postpartum Period] explode all trees | 2803 |
| #3 | After delivery | 35999 |
| #4 | Immediate postpartum | 1103 |
| #5 | #1 OR #2 OR #3 OR #4 | 47880 |
| #6 | MeSH descriptor: [Long-Acting Reversible Contraception] explode all trees | 51 |
| #7 | Long-Acting Reversible contraceptive | 360 |
| #8 | IUCD | 123 |
| #9 | IUD | 2776 |
| #10 | Intrauterine contraceptive device | 1620 |
| #11 | Implant | 18968 |
| #12 | #6 OR #7 OR #8 OR #9 OR #10 OR #11 | 22369 |
| #13 | Uptake | 25876 |
| #14 | Use | 631997 |
| #15 | Acceptance | 20424 |
| #16 | Adoption | 7151 |
| #17 | #13 OR #14 OR #15 OR #16 | 664432 |
| #18 | Ethiopia | 1850 |
| #19 | MeSH descriptor: [Ethiopia] explode all trees | 686 |
| #20 | #18 OR #19 | 1850 |
| #21 | #5 AND #12 AND #17 AND #20 | 8 |
