# Supplementary material for: Immediate postpartum long-acting reversible contraception in Ethiopia: A scoping review
Source: PLoS One. 2026 Jul 6;21(7):e0352352. doi: 10.1371/journal.pone.0352352 (PMC13336211; doi:10.1371/journal.pone.0352352)
Supplement: S1 Table — (DOCX) [file pone.0352352.s003.docx]

**S1 table. Characteristics of Included Studies on IPP-LARC in Ethiopia**

| **Author name & Year** | **Study Region** | **Objective** | **Setting** | **SD** | **SS & P** | **TM** | **Method type** | **IPP-LARC (%)** | **Determinants, Recommendations & Limitations**  **/Gaps** |
| --- | --- | --- | --- | --- | --- | --- | --- | --- | --- |
| Arero et al., 2022 [23] | Oromia | To assess the prevalence and associated factors of LARC methods utilization among counseled mothers in the immediate postpartum period | PH | CSS | 393 PMs | ≤48 hrs | IUCD + Implant | 53.2% (I: 47.4% and IUCD:5.8%). | Factors: higher income, having >4 children, planning to delay pregnancy >2 years, no fertility desire, prior LARC use, counseling during ANC. ***Recommendation:*** Emphasizes need to integrate LARC counseling into continuum of care from ANC.  **Limitation**: Institution-based; timing of counseling suboptimal; convenience sampling. |
| Usso et al., 2021 [21] | Oromia | To assess utilization of IPP-LARC and identify associated factors among women who gave birth in selected public health facilities | PHF | CSS | 530 PMs | ≤48 hrs | IUCD + Implant | 18.5%(I:5.7% and IUCD:12.8%). | Key Associations: Positive: Discussion with partner, Postpartum counselling. Negative: Distance >30 mins, Disrespect/abuse during childbirth. ***Recommendation:*** Provide respectful maternity care. Strengthen FP counselling. Involve male partners. Support women living far.  **Limitation**: Stressful postpartum period; Male consent barrier; Focus only on LARC. |
| Tegene et al., 2025 [37] | Oromia | To determine the magnitude and factors associated with IPP-LARC utilization among women who gave birth at AHMC | PH | CSS | 421 PMs | ≤48 hrs | IUCD + Implant | 43.9% (I:42.0% and IUCD:1.9%) | Planning to delay next pregnancy by ≥2 years, FP counselling during ANC, Partner involvement in counselling, Previous history of LARC use. ***Recommendation:*** Integrate FP counselling during ANC. Involve partners during counselling sessions**.Limitation**: Cross-sectional design; Single-institution study. |
| Wudineh et al., 2023 [22] | Addis Ababa | To assess the utilization of IPPLARC and its associated factors among mothers who delivered in selected public hospitals | PHF | CSS | 417 PMs | ≤48 hrs | IUCD + Implant | 30.7%(I:23.3% and IUCD:7.4%) | Factors: Age 25-34 years, Positive attitude, Discussion with partner, Counselling during ANC. ***Recommendation:*** Healthcare providers should clarify myths; Encourage discussion with partners and counselling during ANC. **Limitation:** Institutional-based; Cross-sectional design. |
| Gebremichael, 2019 [52] | Addis Ababa | To measure the magnitude and identify factors associated with IPP-LARC use during the immediate postpartum period | PHF | CSS | 693 postpartum mothers | ≤48 hrs | IUCD + Implant | 17.5%. | Factors: mother’s attitude, husband’s attitude, Place of delivery, prior IUCD knowledge. ***Recommendation:*** Integrate FP counseling with ANC; enhance maternal and partner awareness.**Limitation:** Limited to Addis Ababa public facilities. |
| Teshome et al., 2025 [39] | Oromia | To determine the prevalence of IPP-LARC uptake and identify associated factors | PH | CSS | 317 PMs | ≤48 hrs | IUCD + Implant | 35.3% (I:30.3% and IUCD:5.0%). | Factors: Having ≥4 children, Cesarean delivery, prior knowledge, previous LARC use, contraceptive counselling, partner discussion. ***Recommendation****:* Integrate structured counselling; involve male partners; targeted awareness campaigns.Limitation: Single-hospital; Cross-sectional; Recall and social desirability bias. |
| Ayane et al., 2023 60] | Addis Ababa | To assess magnitude & factors assoc. with IPPLARC utilization | PHF | CSS | 411 PMs | ≤48 hrs | IUCD + Implant | 27.3%(I:22.4% and IUCD:4.9%). | Factors: Discussion with partner, partner support, good knowledge, favorable attitude. ***Recommendation***: Strengthen partner support & discussion; clarify myths about side effects. **Limitation:** Institution-based, cannot establish causality, potential social desirability bias. |
| Belayihun et al., 2021 [18] | Amhara, Oromia, Tigray, SNNP | To identify factors influencing LARC use during immediate postpartum among women who delivered in health facilities. | HC | CSS | 884 PMs | ≤12 months | IUCD + Implant | 36.3%9I:29.1% and IUCD:7.2%). | Key predictors: FP information, counseling, maternity waiting home stay, Child immunization.  ***Recommendation****:* Integrate PPFP with maternal and child health services; reinforce counseling. **Limitation**: Did not explore qualitative aspects; recall bias; limited to women delivering in facilities. |
| Gudeta et al., 2025 [25] | Central Ethiopia | .  To assess the level of IPP- LARC utilization and associated factors | CB | CCSS | 836 PMs | ≤ 6 monts | IUCD + Implant | 18.3%(I:5.6% and IUCD:2.7%). | Factors: Secondary education, Wealthier families, Health facility delivery, Higher women empowerment, Favorable attitude, FP Counseling during ANC. ***Recommendation***: Improve quality of FP counseling; community education; empower women. **Limitation:** Cross-sectional design; potential recall and social desirability bias |
| Silesh et al., 2022 [58] | Amhara | To assess the prevalence of immediate postpartum family planning (IPPFP) utilisation and associated factors | PH | CSS | 394 PMs | ≤48 hrs | IUCD + Implant | 20.0% (I:17.2% and IUCD:2.8%). | Factors: Maternal age (30-34), planned pregnancy, undecided reproductive intention, partner support, positive attitude, satisfaction with intrapartum care. ***Recommendation:*** Strengthen community awareness; promote partner involvement; ensure maternal satisfaction with care. **Limitation**: Cross-sectional design; Facility-based; Did not address provider-related factors; Social desirability bias. |
| Tariku et al., 2022 [20] | Sidama | To assess the uptake of IPP-LARC methods and its associated factors among women who gave birth at HUCSH | PH | CSS | 418 PMs | ≤48 hrs | IUCD + Implant | 25.4%(I:19.4% and IUCD:6.0%). | Reasons for non-use: preference to start after 6 weeks, desire other methods, husband's influence. Factors: Unplanned birth, Postpartum counselling on LARC. ***Recommendation***: Strengthening FP counselling during immediate postpartum period. Further study on provider-related factors. |
| Demissie et al., 2019 [46] | Addis Ababa | To determine IPPFP utilization and associated factors among women attending delivery | PHF | CSS | 586 PMs | ≤48 hrs | IUCD + Implant | 9.9%(I:6.2 and IUCD:3.7%). | Factors: Good Knowledge, Favorable Attitude, Counseled at delivery room. ***Recommendation:*** Strengthen counseling at delivery, provide IEC, train health extension workers, awareness campaigns. **Limitation**: Cross-sectional design; program-related barriers not assessed; suggests qualitative research. |
| Belay et al.2018 [44] | Addis Ababa | To assess the barriers to uptake of long-term and permanent FP methods among immediate postpartum mothers | PH | CSS | 422 PMs | ≤48 hrs | IUCD + Implant | 44.3%(I:36.5% and IUCD:7.8%. | LARC utilization=36%. Key predictors: FP information, counseling, maternity waiting home stay, Child immunization. Recommendation: Integrate PPFP with maternal and child health services; reinforce counseling.**Limitation:** Did not explore qualitative aspects; recall bias; limited to women delivering in facilities. |
| Adella et al., 2024 [61] | Amhara | To determine factors influencing utilization of LARC immediately after childbirth. | PHF | CCS | 324 PMs | ≤48 hrs | IUCD + Implant | N/A | Determinants: secondary education or higher, ANC attendance, no desire for more children, counseling, good knowledge. ***Recommendation****:* Enhance maternal education, Counseling during ANC and postpartum, and strengthen LARC awareness. Limitation: Limited to public facilities; potential recall bias; cross-sectional nature limits causality. |
| Aemro et al., 2022 [17] | Oromia | To assess utilization of IPP-IUCD and associated factors among women who gave birth in public hospitals | PHF | CSS | 493 PMs | ≤48 hrs | IUCD | 22.1% | Factors: ≥3 children, No desire for more children, Counseling after delivery, and good knowledge.Limitation: Conducted only in public facilities and limited to immediate postpartum (48h); excludes private clinics. |
| Melkie et al., 2021 [55] | Amhara | To determine the utilization and factors associated with an IPPIUCD | PH | CSS | 423 PMs | ≤48 hrs | IUCD | 4.02%. | Factors: Age 35-49, college and above education, receiving IPP-IUCD counseling, desire for birth spacing >36 months. Reason for Non-Use: Couple refusal, fear of side effects. ***Recommendation:*** Encouraging women's education and informing health professionals; emphasis on counseling. |
| Dinsa et al.,2024 [47] | Oromia | To assess the determinants of immediate uptake of postpartum PPIUCD among women who delivered in hospitals. | PHF | CSS | 290 PMs | ≤48 hrs | IUCD | 19.3%. | Factors: Receiving PPIUCD counseling, early ANC initiation, planned future pregnancy, knowledge. ***Recommendation:*** Healthcare program managers should develop strategies enhance PPIUCD. **Limitation**: Facility-Based; Cross-Sectional Design; No Follow-up. |
| Geda et al., 2021 [19] | Addis Ababa | To assess immediate postpartum PPIUCD utilization and its influencing factors | PH | CSS | 286 PMs | ≤48 hrs | IUCD | 26.6%. | Factors: Good knowledge, receiving PPIUCD counseling, Spousal discussion increased odds. Being a housewife, needing partner approval decreased odds. ***Recommendation***: Empower women through information. Encourage spousal discussion. Integrate PNC with PPFP counseling. **Limitation:** Conducted only in capital city's public hospitals; cannot establish causal relationships. |
| Guye et a., 2023 [51] | Oromia | To assess the utilization of an IPPIUD and its associated factors among women who gave birth in public hospitals | PH | CSS | 599 PMs | ≤48 hrs | IUCD | **27.2%** | Factors: Age 25-34, Early ANC (≤16 weeks), Planned pregnancy, Adequate knowledge, Favorable attitude, Counseling. ***Recommendation:*** Integrate IPPIUD services into routine MCH, provide awareness through ANC counseling.Limitation: Social desirability bias, excluded non-hospital births. |
| Tefera et al., 2017 [36] | Sidama | To assess IPP-IUCD utilization and its associated factors among mothers who delivered in selected facilitie | PH | CSS | 310 PMs | ≤48 hrs | IUCD | **21.6%.** | Factors: no future childbearing plan, undecided, ever hearing, receiving counselling. ***Recommendation:*** Integrate standard PPIUD counselling; Raise public awareness via media. Limitation: Cross-sectional; Limited facilities; Recall bias. |
| Abdullahi et al., 2024 [69] | Addis Ababa | To assess the utilization and factors associated with the uptake of IPP-IUD | PH | CSS | 412 PMs | ≤48 hrs | IUCD | 11.0% | Factors: Parity, Mode of Delivery. Main Barrier: Fear and concern (61.6%). ***Recommendation:*** Address gaps in awareness and misconceptions; public education. Recommendation: Integrate FP counselling; strengthen community awareness; promote partner involvement. Limitation: Institutional-based in capital; not nationally representative. |
| Hagos et al., 2020 [24] | Amhara | To assess IPP-IUCD utilization and associated factors among mothers who delivered at the hospital | PH | CSS | 182 PMs | ≤48 hrs | IUCD | 3.3% | Key factors: acceptance, reasons for refusal: Fear of side effect  ***Recommendation:*** Correct misconceptions through counseling; supervision; bridge acceptance-utilization gap. **Limitation:** Cross-sectional; Facility-based; Did not deeply explore knowledge/attitude. |
| Assefaw et al., 2021 [62] | Amhara | To identify determinants of IPP IUCD uptak | PH | CCS | 420 PMs | ≤48 hrs | IUCD | N/A | Completed secondary education, having 3-4 & ≥5 children, attending 3 ANC visits, ever hearing about PPIUCD, receiving counselling. ***Recommendation:*** Advise women to follow ANC, improve access to information, provide counselling. |
| Mohammed et al., 2020 [62] | Southern Ethiopia region | To assess determinants of IPPIUCD utilization among mothers | PHF | CCS | 510 PMs | ≤48 hrs | IUCD | N/A | Key Determinants: Partner Support, Birth Interval, Fertility Plan, Counseling during early labor. ***Recommendation:*** Counsel both partners during ANC; integrate PPIUCD with MCH; mobilize male involvement. **Limitation:** Facility-based; potential for bias. |
| Sium et al., 2022 [65] | Addis Ababa | To determine the impact of a dedicated obstetrics and gynecology resident for postpartum family planning counselling on uptake of IPPLARC | PH | Pre-post observational study | 1,684(Pre-intervention:908 and post-intrvention:776 deliveries) | ≤48 hrs | IUCD | **N/A** | **IUCD Uptake: 20.4% (with resident) vs 15.4% (without).** Factors: Delivery month with dedicated resident, Mode of delivery. ***Recommendation:*** Assign a dedicated care provider for PPFP counselling before discharge. **Limitation**: Short observation time; did not control for known factors. |
| Wayessa et al., 2020 [64] | Oromia | To assess the effectiveness of focused family planning counseling in increasing IPPIUCD uptake | PHF | QES | 726 PMs | ≤48 hrs | IUCD | **N/A** | **IUCD Uptake**: Intervention (12.4%) vs. Non-intervention (4.8%). FFPC strongest predictor (AOR: 5.92). Other factors: marital status, education, gravidity, decision-making, FP knowledge. ***Recommendation:*** Revise and scale up focused, theory-based FP counseling. Limitation: Quasi-experimental; Social desirability bias; Not blinded. |
| Sori et al., 2023 [29] | Oromia | To assess the effect of a Continuous Quality Improvement (CQI) strategy using the Plan-DoStudy-Act (PDSA) model to increase IPP-LARC use | PH | CQI | 789 (BL), 820(I) PMs & Providers | ≤48 hrs | IUCD + Implant | **N/A** | LARC Utilization increased from 6.9% to 25.4%. Key Findings: Training, commodity availability, audit/feedback. Barriers: Fear, husband disapproval, lack of staff, commodities. **Recommendation**: Sustain CQI activities; Ensure commodity availability; Test change ideas in other facilities. Limitation: Training did not include all staff; No control group; No long-term follow-up. |
| Tesfaye et al., 2023 66] | Addis Ababa | To assess the combined effect of a package of interventions on the use and quality of IPP-FP services | PH | Pre-post intervention | 421(pre-intervention:226 and Post-interventio:195)PMs, 10 HWs | ≤48 hrs | IUCD + Implant | **N/A** | LARC uptake: Increased from 65.9% to 72.3%. ***Recommendation:*** Scale up quality improvement interventions like private counseling spaces and provider training. Limitation: Lack of a control group |
| Abasimel et al., 2024 [38] | Oromia | To determine the acceptance level and associated factors of an IPPIUCD | PH | CSS | 534 PMs | ≤48 hrs | IUCD | 17.8% | Factors: maternal occupation (Government), knowledge, favorable attitude. Main Reasons for Rejection: Not preferred method, fear of side effects. ***Recommendation****:* Strengthen health education, attitude change interventions, and regular training for healthcare workers. |
| Gebremedhin et al., 2021 [46] | Southern Ethiopia | To assess acceptability and factors associated with IPP-IUCD use | PH | CSS | 452 PMs | ≤48 hrs | IUCD | 35.6%. | Factors: Multiparity, Completed ANC follow-up, Received Counseling, Prior discussion with partner. ***Recommendation***: Integrate PPIUCD counseling throughout pregnancy care (ANC, delivery, PNC). **Limitation**: Facility-based; Reliance on self-reported data. |
| Geberemariam et al., 2024 [48] | Addis Ababa | To assess the acceptance of immediate PPIUCD and associated factors among women giving birth | PH | CSS | 336 PMs | ≤48 hrs | IUCD | 12.5%. | Factors: Private employee, Daily Laborer, Student, Monthly Income >7,901 ETB. ***Recommendation:*** Launch public awareness campaigns; train healthcare workers; target interventions toward housewives and low-income women.Limitation: Cross-sectional design limits causal inference; potential for social desirability bias; limited to public hospitals in the capital city. |
| Samuel et al., 2023 33] | Sidama | To determine the magnitude of informed choice and its associated factors among women receiving IPP-LARC | PH | CSS | 373 PMs | ≤48 hrs | IUCD + Implant | 23.5% | Factors: Exposure to FP posters, PPFP counselling during ANC, Prior contraceptive use, Secondary education, Higher education. ***Recommendation:*** Strengthen counseling during ANC and before delivery to increase postpartum LARC uptake.Limitation: Potential for courtesy bias; Cross-sectional design limits causal inference; Gap in quality of counseling. |
| Shiferaw et al., 2023 [57] | Sidama | To assess acceptance, utilization, and factors associated with IPPIUCD | PHF | CSS | 382 PMs | ≤48 hrs | IUCD | **16.4%. and 10.0%** | Factors of acceptance: Counseling, positive attitude, short birth interval, No plan for more children. Factors utilization: Husband support, Daytime delivery, Having ≥3 children. ***Recommendation:*** Provide routine PP-IUCD counseling; Implement couple-based FP; Ensure FP services available day and night.Limitation: Cross-sectional design; Facility-based; Suggests qualitative research. |
| Gonie et al., 2018 50] | Oromia | To determine the level of acceptability and factors associated with IPP-IUCD use | PHF | CSS | 429 PMs | ≤48 hrs | IUCD | 12.4%. | Factors: Completed secondary education, attending 3 ANC visits. **Limitation:** Facility-based; Short counselling-to-decision time. |
| Kefeni et al. ,2025 54] | Oromia | To assess acceptance rate of IPP IUCD and associated facto | PH | CSS | 373 PMs | ≤48 hrs | IUCD | 9.9%. | Factors: Mode of delivery, Number of pregnancies, Partner occupation. ***Recommendation:*** Community-wide education and qualitative studies to address fears. Limitation: Only public hospitals; no qualitative data. |
| Daba et al., 2021 45] | Oromia | To assess the intention to use postpartum PPIUCD and associated factors among pregnant women attending antenatal clinics | PHF | CSS | 417 PW | ANC contact | IUCD | 34.9%. | Factors: age, education, occupation, income, knowledge. ***Recommendation:*** Improve awareness; integrate PPIUCD counseling into ANC and FP programs. Limitation: Limited to public institutions; cross-sectional design. |
| Tefere et al., 2023 [36] | Oromia | To assess intention and barriers to use IPP-IUCD among pregnant women attending ANC | PHF | CSS | 314 PW | ANC contact | IUCD | 37.6% | . Predictors: education, knowledge, previous use, parity. Barriers: worry for health, satisfied with previous method. ***Recommendation:*** Strengthen provider counseling and education; address barriers like fear and partner opposition. Limitation: Excluded husband’s influence; cross-sectional; recall bias. |
| Sisay et al., 2023 [59] | Amhara region | To assess knowledge, attitude, and associated factors toward postpartum PPIUCD among pregnant women | PHF | CSS | 417 PW | ANC contact | IIUD | 36% and 48.7% | Factors: occupation, residence, discussion, education, ANC visits. ***Recommendation:*** Enhance women's education, encourage ANC, provide effective IUCD counseling, community awareness. Limitation: Cross-sectional design; social desirability and recall bias; limited generalizability. |
| Meskele et al., 2024 [67] | Southern Ethiopia | To explore barriers and enablers to the implementation of immediate postpartum and post-abortion family planning (IPPFP & PAFP) service integration | HC | QS | 41 IDIs/KIIs, 6 FGDs (PMs, Husbands, Health care provider | Multiple | IUCD + Implant | N/A | Barriers: Misconceptions, husband's decision-making, cultural/religious barriers, staff shortages, supply delays. Enablers: Community structures, equal access, waived costs, collaboration. ***Recommendation:*** Implement high-impact interventions: target male partners and adolescents, ensure supplies, build staff capacity. Limitation: Context-specific; Researcher bias; Excluded adolescents/post-abortion women. |
| Hailegorgi et al., 2024 [53] | Southern Ethiopia | To assess the prevalence and factors associated with the intention to use IPP-LARCs among third-trimester pregnant women | CB | CSS | 372 PW | ANC contact | IUCD + Implant | 35.6%. | Factors: Direct Attitude, Direct Subjective Norm, Direct Perceived Behavioural Control, History of LARC use, Age. ***Recommendation:*** Address misconceptions via SBCC. Build provider capacity. Leverage ANC, peers, social media. Engage partners. Limitation: Cross-sectional; Social desirability bias; Context-specific beliefs. |
| Geda et al., 2022 [68] | Nationwide | To determine the pooled magnitude and factors influencing IPP-IUCD use in Ethiopia | Multiple | SR &MA | 7 studies | N/A | IUCD | 8.37%. | Predictors: age, awareness, knowledge, counseling, birth interval. ***Recommendation:*** Strengthen counseling, especially for women <25; integrate IPPIUCD with postpartum services. Limiitation: Limited regional coverage; high heterogeneity. |
| Mickler et al., 2021 (64) | Addis Ababa, Afar, Oromia, Amhara, SNNPR, Tigray | To examine if women's receipt of IPP-FP counseling varied by individual and facility-level characteristics | Multiple | CCSS | 936 PMs & 224  facilities | Secondary data | Receipt of IPP-FP counseling | 27.0% | Factors: parity, delivery mode, provider type, prior contraceptive use. ***Recommendation:*** Ensure equitable, high-quality IPPFP counseling for all women. **Limitation:** Facilities not fully representative; Could not assess quality; Need to understand provider barriers. |

***Keys:*** *CCSS: Community based cross-sectional study, CSS: Facility based cross-sectional study, CCS: Case-control study, CQI: continues quality improvement intervention, PH: Public hospitals, PHF: puplic health facilities, HC: health centers, PMs: postpartum mothers PW: pregnant women****,*** *QI: quality improvement intervention, QES: Quasi-experimental study, SS: Sample size, SD: study design, TM:Time of measurement.*
