# Supplementary material for: Immediate postpartum long-acting reversible contraception in Ethiopia: A scoping review
Source: PLoS One. 2026 Jul 6;21(7):e0352352. doi: 10.1371/journal.pone.0352352 (PMC13336211; doi:10.1371/journal.pone.0352352)
Supplement: S2 Table — (DOCX) [file pone.0352352.s004.docx]

**S2 table. Comparison of IPP-LARC Uptake in Intervention Studies**

| Study (Author, Year) | Intervention Type | Baseline Uptake | Post-Intervention Uptake | Change |
| --- | --- | --- | --- | --- |
| Sori et al. (2022) | CQI (PDSA Cycle) | 6.9% | 25.4% | +18.5% |
| Wayessa et al. (2020) | Health Belief Model Counseling | 4.8% | 12.4% | 7.6% |
| Tesfaye et al. (2023) | Multi-component Package | 65.9% | 72.3% | 6.4% |
| Sium et al. (2022) | Dedicated staff/Resident | 15.4%% | 20.4% | 5.0% |
